# Visual activation of extra-striate cortex in the absence of V1 activation

**DOI:** 10.1016/j.neuropsychologia.2010.10.022

**Published:** 2010-12

**Authors:** Holly Bridge, Stephen L. Hicks, Jingyi Xie, Thomas W. Okell, Sabira Mannan, Iona Alexander, Alan Cowey, Christopher Kennard

**Affiliations:** a FMRIB Centre, University of Oxford, John Radcliffe Hospital, Oxford OX3 9DU, United Kingdom; b Department of Clinical Neurology, University of Oxford, John Radcliffe Hospital, Oxford OX3 9DU, United Kingdom; c Department of Experimental Psychology, University of Oxford, Parks Road, Oxford OX1 3UD, United Kingdom

**Keywords:** Primary visual cortex, Blind, fMRI, Lateral geniculate nucleus, Structural imaging

## Abstract

When the primary visual cortex (V1) is damaged, there are a number of alternative pathways that can carry visual information from the eyes to extrastriate visual areas. Damage to the visual cortex from trauma or infarct is often unilateral, extensive and includes gray matter and white matter tracts, which can disrupt other routes to residual visual function. We report an unusual young patient, SBR, who has bilateral damage to the gray matter of V1, sparing the adjacent white matter and surrounding visual areas. Using functional magnetic resonance imaging (fMRI), we show that area MT+/V5 is activated bilaterally to visual stimulation, while no significant activity could be measured in V1. Additionally, the white matter tracts between the lateral geniculate nucleus (LGN) and V1 appear to show some degeneration, while the tracts between LGN and MT+/V5 do not differ from controls. Furthermore, the bilateral nature of the damage suggests that residual visual capacity does not result from strengthened interhemispheric connections. The very specific lesion in SBR suggests that the ipsilateral connection between LGN and MT+/V5 may be important for residual visual function in the presence of damage to V1.

## Research highlights

▶ Gray matter of V1 shows abnormal T1 characteristics and its perfusion is reduced. ▶ Damage is confined to gray matter with no adjacent white matter involvement. ▶ BOLD activation levels in the calcarine sulcus are drastically reduced. ▶ Activation of extrastriate regions to visual stimulation is preserved. ▶ Pathway between LGN and V1 shows degeneration; between LGN and V5/MT is intact.

## 1 Introduction

Damage to the primary visual cortex (V1) in primates removes the major visual input to the cortex from the corresponding part of the retina. Such damage is usually unilateral and caused by ischemic events or traumatic head injury. Many such patients retain some residual visual sensitivity, but without conscious visual perception, in their otherwise blind field defect, i.e. ‘blindsight’. Other patients appear to maintain some residual, but degraded, conscious vision and it is not clear whether the mechanisms are part of the same spectrum (Blythe, Kennard, & Ruddock, 1987) or arise from distinct pathways (Weiskrantz, Barbur, & Sahraie, 1995). There is evidence, when the damage to V1 is unilateral, of an increase in transcallosal connectivity (Bridge, Thomas, Jbabdi, & Cowey, 2008; Silvanto, Walsh, & Cowey, 2009).

When damage is bilateral, the regions of normal vision are further reduced, and the patient is severely visually impaired. The few cases of bilateral damage that have been described thoroughly, with neuroimaging used to verify the damage, indicate a puzzling variety of outcomes. Where damage occurred at birth or a young age, visual performance appears to fare better, particularly when the damage is restricted to V1. In a case where damage appeared to be limited to V1, a child who had appeared blind at 2.5 years had improved basic visual function by 7 years, and no longer seemed visually impaired (Bova et al., 2008). In contrast the subject with extensive bilateral occipital damage from birth studied by Giaschi et al. (2003) was severely visually impaired, although he could name a few colors and perceive rapid movements. Functional MRI activation patterns to the moving stimuli, however, were located predominantly in the posterior superior temporal sulcus bilaterally; there was no occipital activation. Where damage occurs in adulthood, it appears that the effects are significantly worse, with no cortical activation to visual stimuli, and little evidence of visual function (de Gelder et al., 2008).

Here we present a young adult patient (SBR) who has bilateral damage to V1, with sparing of V1 only at the anterior tip of the calcarine sulcus, corresponding to far peripheral vision. He shows decreased integrity of the gray matter in the calcarine sulcus, with no damage to the adjacent white matter. Furthermore, fMRI reveals significant levels of BOLD activation to high contrast visual stimuli in extrastriate cortex (predominantly MT+/V5), but scant evidence of activation in V1. Since the V1 damage is bilateral, this extra-striate activation cannot be explained by interhemispheric transfer of visual information. When eccentrically presented stimuli, corresponding to regions of spared V1, are presented the extent of extrastriate cortex activation is increased.

## 2 Materials and methods

### 2.1 Subjects

The study was conducted under ethical approval from the Oxfordshire NHS Research Ethics Committee (08/H0605/156), and all subjects provided informed written consent. Five age-matched controls were used for the diffusion imaging (mean age 21.8 ± 2.5), the gray matter analysis (mean age 21.8 ± 2.5) and the perfusion imaging (mean age 23.6 ± 5.1). Three subjects were used for the fMRI data analysis (mean 24 ± 6.9).

### 2.2 Patient SBR

SBR is a university educated professional, who suffered a hypoxic event of unknown origin at the age of 22, which led to a 3-month period in a coma. On awaking, he was clinically blind and has recovered little sight in the 2 intervening years. Clinical MRI showed polar occipital infarcts with altered flow in the left vertebral artery suggesting possible previous vascular disturbance, probably a dissection. His residual conscious vision appears restricted to a crescent in the upper visual field. The patient undertook 3 scanning sessions. The first acquired structural and diffusion-weighted images and the other sessions consisted of fMRI data acquisition and perfusion imaging. Scanning was performed at 26, 30 and 32 months post event.

### 2.3 Psychophysics

(i) Gabor patches (standard deviation = 2.5° or 0.75°), spatial frequency 1 cycle/deg and temporal frequency of 5 Hz were presented for 2 s. The larger patch was presented at a retinal eccentricity of 45°, and the smaller at 8° and 16°. Using a temporal two alternative forced-choice paradigm, the patient indicated verbally (if necessary by guessing), which interval contained the Gabor patch, presented along one of the four diagonals. Fixation was monitored continuously using an Eyelink 1000 Eyetracker.

In the remaining three psychophysical investigations, fixation throughout was ensured by asking SBR to look at his finger which rested against the fixation mark, even if he could not see the finger. His arm did not obscure the display. Stimuli were presented in the center of the screen, but the fixation mark was positioned either 4 cm below the lower edge of the stimulus, or 4 cm above the upper edge in order for the stimulus to be presented to the upper or lower visual field respectively.

(ii) To assess SBR's color discrimination, blue or yellow stimuli were used as SBR was a protonomolous trichromat before his injury. Stimuli were presented on a 2.5-cdm^2^ background on a 17-inch color monitor incorporating a touch screen (Phillips UP2799) at a viewing distance of 28 cm. The stimulus, a blue (5.5 cdm^2^) or yellow (5.5 cdm^2^) 5 cm × 5 cm patch was presented to the upper visual field (as described above). SBR completed 1 block of 100 trials and stimuli were equiprobable. The subject sat in a dimly lit room with his head supported and trials were initiated by the experimenter saying ‘now’ followed by a 200-ms stimulus which SBR named as Blue or Yellow. Results were recorded manually.

(iii) Motion stimuli were generated by a Toshiba laptop computer with a 75-MHz Pentium Processor and a standard SVGA video card with 6-bit gray resolution. They were displayed with gamma correction on a Dan Monitor at a non-interlaced frame rate of 80 Hz. Moving stimuli were presented at speeds of 4 or 32°/s to SBR's upper visual field. Each block contained 20 trials and SBR completed four blocks for each condition. The stimulus was a random dot kinematogram with 100% coherence in a circular 20° window cut out of a sheet of black card placed over the VDU. The window contained on average 30 one-degree white spots on a gray background. Each spot was 10 cd/m^2^ and the luminance contrast with the background was 1.0. The component spots had an infinite lifetime, meaning that every spot moved smoothly throughout each trial. Trials were initiated by the experimenter by saying ‘now’ and simultaneously pressing a key on the keyboard. The coherent global movement was always to the right. Each trial consisted of two 300-ms temporal intervals separated by 20 ms and each interval was marked by a 50-ms tone, and a tone one octave higher indicated the end of the second interval. SBR verbally reported whether the stimulus moved in the first or the second interval and the experimenter recorded the appropriate response on the keyboard. SBR initially carried out a practice block in order to determine that he understood the instructions and knew the task before the fixation was moved to the top of the screen to ensure the display was in SBRs lower, blind, field.

(iv) In the localization and detection tasks, stimuli were generated using a Cambridge Research Systems VSG 2/5, programmed with a Dan computer and visual basic software. Stimuli were displayed on an EIZO 19 inch monitor, calibrated using OPTICAL (Cambridge Research Systems), with a viewing distance of 57 cm. The stimulus was a plain 10 × 10° white square with a contrast of 0.3 in the localization task or with a contrast of either 1.0 or 0.2 in the present/absent task. The background was always 10 cdm^2^. Each block of one stimulus type contained 30 trials and SBR completed two blocks of each stimulus condition, in his lower visual field, reporting whether the stimulus appeared on the left or right of the screen. The experimenter started each trial with a key press and saying ‘now’, which was instantaneously followed by a 200 ms stimulus in either the bottom left or right of the VDU, Stimuli were equiprobable. The experimenter recorded SBR's verbal response on the keyboard. In the localization task SBR indicated whether the stimuli appeared on the left or right of the screen in his lower field. Again stimuli were equiprobable. In the present/absent task the stimuli were presented on only half the trials at random and SBR categorized them as present or absent. After each block of trials SBR was asked whether he had experienced any kind of awareness of the stimuli.

### 2.4 MR imaging

Scanning was performed at the Oxford Centre for Clinical Magnetic Resonance using a Siemens 3T Trio scanner with a 12-channel head coil. A 1 mm × 1 mm × 1 mm T1 structural scan was performed using standard parameters (magnetization prepared rapid gradient echo (MPRAGE), repetition time (TR) = 15 ms, echo time (TE) = 6.0 ms). Diffusion-weighted images were acquired axially using echoplanar imaging, with isotropic voxels of 2 mm^3^. The diffusion weighting was isotropically distributed through space (Jones et al., 1999) along 60 directions using a *b*-value of 1000 s/mm^2^. Two sets of diffusion-weighted data were collected, and for each set five volumes with no diffusion weighting were acquired during the sequence.

Functional MRI consisted of 2 repeats of 4 different scans. Three scans consisted of an 8-Hz counterphase flashing black and white checkerboard (2.0° checks), 12 × 24 contrasted with a mid-gray fixation screen. In the first scan the checkerboard was presented centrally (extending 6° eccentrically) in SBR only. In the subsequent scans, subjects were instructed to fixate on a spot either the top or bottom of the screen with the same size checkerboard placed 4° from the fixation point (extending to 16°). A ‘motion’ scan designed to activate area MT+/V5 contrasted moving dots with stationary dots in a block design. An 8° diameter patch of white square dots (0.5 × 0.5°) was presented on a black background. During the moving block, the dots moved radially at a speed of 30°/s, reversing direction every second. In each case a complete cycle of stimulus on and off took 30 s and was presented 8 times (TR = 3 s; 2 mm x 2 mm x 2 mm). While SBR could not necessarily see the fixation spot, he was able to direct his eyes up, down or central based on his detecting the intensity difference at the edges of the screen.

### 2.5 Perfusion imaging

A pseudo-continuous arterial spin labeling (ASL) approach was used with a labeling duration of 1.5 s and a post labeling delay of 1 s (similar to the non-selective cycles described in Okell et al., 2010). Background suppression of static tissue was achieved using two non-selective inversion pulses with inversion times chosen within timing constraints to null tissues with a T1 at 390 or 780 ms. A gradient-echo echo-planar imaging (GRE-EPI) readout was used, with TR/TE = 4660 ms/13 ms. Twelve axial slices (3 mm^3^ voxels, 0.6 mm gap) were prescribed for each subject, providing full coverage of the calcarine sulcus. 50 tag/control pairs were collected on each subject, giving a total experiment time of ∼8 min.

### 2.6 Data analysis

Structural data analysis consisted of defining regions of the anterior and posterior calcarine sulcus and the intraparietal sulcus (IPS). Arbitrary intensity values were extracted from each of these gray matter masks to determine the mean intensity values. To determine whether SBR's data lay within the control group data, a *z*-statistic was calculated using the following equation *z* = (SBR value − mean control value)/control standard deviation. His data were considered significantly different from control subjects if |*z*| > 2.

Functional data were analyzed using FMRIB's easy analysis tool (FEAT), part of the FSL toolbox (www.fmrib.ox.ac.uk). Images were pre-processed using a number of steps: head movement correction using MCFLIRT, spatial smoothing with a Gaussian kernel full-width, half-height (FWHM) = 5 mm, mean-based intensity normalization, non-linear high-pass temporal frequency filtering (Gaussian-weighted straight line fitting, with sigma = 30 s). Data from the two runs of each stimulus were combined using a fixed effects analysis.

To perform probabilistic tractography, masks for the lateral geniculate nucleus (LGN) and calcarine sulcus (CS) were defined anatomically in MNI space and transformed into the space of each subject. Definitions of area MT+/V5 and dorsal V2/V3 were extracted from the activation data of patient SBR and transformed into the space of the control subjects. Probabilistic tractography produces an estimate of the most likely location of a pathway from a seed point using Bayesian techniques, the details of which have been described elsewhere (Behrens, Berg, Jbabdi, Rushworth, & Woolrich, 2007). A local model for fiber orientation within each voxel is inferred from the data. The probabilisitic tractography consists of drawing pathways by following sample orientations in each voxel along the trajectory. Five thousand sample tracts were generated from each seed voxel within the LGN mask, and only tracts entering the target masks (CS, MT/V5 or dorsal V2/V3) were retained. To ensure that the pathways were restricted in extent an exclusion mask was incorporated to prevent projections anterior to Meyer's loop and crossing into the contralateral hemisphere. Quantitative measures of FA and mean diffusion for individual subjects were extracted by taking the fiber-density-weighted mean within each of the tract.

## 3 Results

### 3.1 Visual function is profoundly impaired

Humphrey and Goldmann perimetry testing both revealed a lack of central vision. SBR's visual fields measured with Goldmann perimetry (classical free-response method) are shown in Fig. 1A, as unstable fixation made Humphrey fields difficult to interpret. There is some sparing in the upper peripheral field, extending slightly into lower peripheral fields. Psychophysical testing with Gabor patches (3° or 10°) indicated that very high contrast (99%) stimuli could be detected throughout the visual field, whereas reducing the contrast to 50% made them undetectable everywhere except the spared regions of space in the upper visual field (Fig. 1B). Detection of the high contrast stimuli could be the result of light scattering. The eccentric position of the center of the stimuli is due to imprecise, yet reliable, fixation of SBR. This performance is superimposed onto the two monocular fields from the perimetry for comparison.

Within his upper field, SBR is also able to detect the onset and color of a blue or yellow patch and identify, 100% correctly, the temporal interval in which a circular patch of random dots (RDK), subtending about 10° moves coherently at either 32 or 4°/s. In contrast, within his lower visual field he was unable to detect the interval in which the dots moved at either speed (48/80 correct). However, in his lower field he scored 83% correct in a task to identify whether a 10°, 200 ms, 20 cd/m^2^ flash on a 10-cd/m^2^ background was in the left or right lower quadrant. When the same stimuli were presented on only half the trials at random and he had to categorize them as present or absent, he scored 53/60 at a contrast of 1.0 (white on black), but saw only 37/60 at a contrast of 0.2. Importantly, even when he scored highly, he reported that he saw nothing and was just guessing, which amounts to blindsight.

### 3.2 V1 shows abnormal tissue characteristics and perfusion

Extraordinarily, the cortical damage that appears to underlie SBR's impaired vision is restricted to the calcarine sulcus (CS), with no damage to adjacent white matter. Fig. 2 shows a T1-weighted structural image of his brain, with the CS indicated by red arrows. The gray matter of this region is of a lower intensity than all other gray matter regions. It is important to note that the values extracted from these images are arbitrary and do not represent ‘real’ T1 values. Nonetheless, such a relative reduction in signal from a T1-weighted image indicates an increase in the water content of the tissue. A quantitative comparison between these values in the posterior CS, IPS and anterior CS can be seen in Fig. 2D, indicating the low T1-weighted signal in the posterior CS in patient SBR compared with controls (*z* > 3.6). In contrast, there was no significant difference between SBR and the control subjects in the other two regions. The apparent sparing of the anterior CS may underlie some of SBR's residual vision, in the upper peripheral visual field and minimally in the lower peripheral field.

In order to investigate whether the abnormal signal from the posterior CS is accompanied by a change in blood flow, a perfusion scan was performed to measure the flow to this region of the sulcus relative to flow in the anterior section and in area MT+/V5. The ratios of perfusion in the posterior CS to anterior CS and area MT+/V5 are shown in Fig. 2E, indicating a relative decrease in perfusion to the posterior CS. The reduction was only significant in the ratio of posterior V1 to MT+/V5 however (*z* = 2.2). Although not a quantitative measure of perfusion, these relative values indicate either a reduction in blood flow to the posterior CS or abnormal time of arrival of the blood.

### 3.3 Activation of the occipital lobe is massively reduced

The functional response of the occipital lobe in SBR to very high contrast visual stimuli is shown in Fig. 3. A central flashing chequerboard produced BOLD activation confined to the human area MT+/V5 and a region dorsal to the CS, presumably corresponding to V2/V3 (A). There is no significant BOLD activity in either the CS or on the inferior surface of the occipital lobe.

The region of the anterior CS that appears unaffected by the insult corresponds to peripheral regions of the visual field. Fig. 3B and C shows the activation in response to a flashing chequerboard that extends up to 25° eccentricity. This increase in stimulus size results in increased activation in the occipital lobe outside of V1, but does not increase the activity in V1 significantly. Data from a control subject can be seen in Supplementary Fig. 1. Not surprisingly this intense visual stimulation produces extremely significant activation throughout the occipital lobe in all control subjects.

There is a large difference in the level of activation between SBR and the control subjects, particularly in the CS where the response of the control subjects is greatest. In area MT+/V5 the response is diminished in SBR relative to controls, presumably due to the lack of input from V1, but the difference is considerably less than for the CS. An additional scan type using 3 runs of radially moving dots compared to stationary dots was also presented to SBR. Again, this stimulus resulted in activation of MT+/V5, but not the CS (data not shown).

### 3.4 Connectivity with the LGN and the retina: retrograde degeneration

The restricted nature of the damage to V1 in SBR means that it is possible to perform tractography between multiple visual areas to investigate the connections that still exist. Tracts were present between LGN and the CS, between LGN and V2 and LGN and MT/V5+, as seen in Fig. 4, left side. These tracts are qualitatively the same as those found in control subjects (data not shown). To establish whether there is any degeneration in these tracts, the mean fractional anisotropy (FA) and mean diffusivity were extracted from the tracts in SBR and the five controls and weighted by the fiber density within the tract (Fig. 4, right side). There is both a significant drop in FA and a significant increase in mean diffusivity in the tract between LGN and V1 (*z* = −2.7 and *z* = 2.6 respectively). There were no significant differences in either FA or mean diffusivity in the tracts between LGN and MT or LGN and V2.

While there may be some degeneration in the optic radiation, there appears to be less effect in the retina. Optical coherence tomography was used to measure the thickness of the retinal ganglion cell layer, using the cross-sectional retinal nerve fiber layer of a circle centered on, but exceeding the size of, the optic disc. These measurements gave a mean thickness of 98.3 μm for the left eye and 96.4 μm for the right eye. Only a single sector in the two eyes showed a borderline thickness, with the others well within normal limits.

## 4 Discussion

The most clear and unusual finding in this rare patient is the restrictive nature of the damage to the brain, with no discernable damage outside of the calcarine sulcus, either in gray or white matter. The activity levels in V1 are extremely attenuated, whereas extra-striate areas are much less affected. Central vision is particularly poor, and centrally presented stimuli do not activate V1 at all. In this case, it seems that activation in extrastriate cortex is initiated through pathways that avoid V1, analogous to the more common unilateral hemianopia. It has been shown that some patients with complete destruction of V1 retain activity in the ispilateral MT+/V5 (Morland, Le, Carroll, Hoffmann, & Pambakian, 2004). It is also true that MT+/V5 contralateral to the lesion is often co-activated, suggesting that ipsilesional MT+/V5 may be influenced by this functioning hemisphere. It is not clear in hemianopic patients whether such contra-lesional activation is due to the ipsilateral representation of space that is known to be present in MST (Huk, Dougherty, & Heeger, 2002), or increased connectivity between MT in the two hemispheres (Bridge et al., 2008). Because of his bilateral damage these signals from the opposite hemisphere are unlikely to be crossing the corpus callosum in SBR, due to his bilateral damage, and therefore the activation of MT+/V5 must be via ipsilateral pathways that avoid V1. A recent study in the macaque monkey by Schmid et al. (2010) highlights the critical role of the LGN in transmitting visual information to the extrastriate cortex when V1 is damaged. Consistent with the behavioral data presented here, the two macaque monkeys with damage to V1 could detect high, but not low contrast stimuli when the LGN was intact. Detection dropped to 0% when the LGN was inactivated.

When more eccentric stimuli are presented, there appears to be some activation of the anterior section of the CS, which corresponds to the far peripheral visual field and is structurally intact when measured both with T1 values and perfusion imaging. In addition to the anterior CS activation, more extensive activation of the ventral visual cortex is also present, suggesting that this region of cortex representing the peripheral regions of the visual field can activate higher visual areas.

The perfusion imaging indicated abnormal blood flow in the posterior region of the calcarine sulcus compared to the flow in area MT+/V5, and in the anterior calcarine. This type of deficit is seen when there is tissue damage, such as in stroke, or in arterial occlusion where blood flow is disrupted. Since the BOLD signal is dependent on the cerebral blood flow, the abnormal perfusion measured here will necessarily impact upon the measured fMRI activation in the calcarine sulcus. Nonetheless, since abnormal perfusion levels often reflect tissue damage, it is unlikely that the tissue has intact neuronal populations, particularly when combined with the abnormal values also measured from the T1-weighted structural images.

Two years after the initial insult, there is some loss of white matter integrity in the optic radiation, indicated by a reduced FA and increase diffusivity in SBR relative to controls. In contrast, the white matter connection between the LGN and MT+/V5 showed FA and mean diffusion values similar to controls. It will be interesting to investigate whether the difference in white matter integrity in these 2 pathways increases over the next few years. The damage to V1 may lead to a strengthening of connections that avoid V1, such as that between LGN and MT+/V5. The lack of V1 activity may cause additional Wallerian degeneration, although the extent of this will depend on whether it more attributable to gray or white matter damage. Almost all lesions of V1 caused by stroke involve some white matter damage, whereas in this case the damage is restricted to gray matter. Longitudinal monitoring of structure and diffusion in SBR's brain may provide some insight into the relative contributions of gray and white matter damage to retrograde degeneration. The reason for the startling restriction of cortical damage to the gray matter of the calcarine sulcus is unclear, but may be related to the well-known rich capillary bed of area V1 compared to the rest of the occipital cortex. If this reflects a difference in metabolic activity in V1 it could make V1 especially vulnerable to the presumed hypoxia that occurred in SBR.

One of the difficult features of this type of case is characterising the residual function, as it can be difficult to control fixation and therefore determine where any stimuli lie in the visual field. The psychophysics presented here indicates conscious perception of very high contrast stimuli, but reduction in the contrast renders SBR unable to perceive them. This is consistent with data from blindsight GY who consciously perceived high contrast targets, but exhibited blindsight at lower contrasts (Weiskrantz et al., 1995).

In cases such as this one, where the patient is both young and highly motivated, the primary concern is to attempt to use the surrounding healthy brain tissue to maximize the residual visual function. Some hope for rehabilitation of visual function can be gained from the experience of the monkey ‘Helen’, who had almost complete striate cortex removal, yet learned to detect and discriminate various stimuli over a period of years (Humphrey, 1974; Humphrey & Weiskrantz, 1967). More recently there have been attempts to improve visual function in patients with unilateral hemianopia, although this is often designed to teach the patient to adapt fixation or saccadic eye movements to recruit the intact hemifield (reviewed in Schofield & Leff, 2009). A combination of exploiting the residual function in the peripheral visual field and strengthening of pathways that bypass V1 may prove to be the most successful strategy for rehabilitation.

## Figures and Tables

**Figure:**
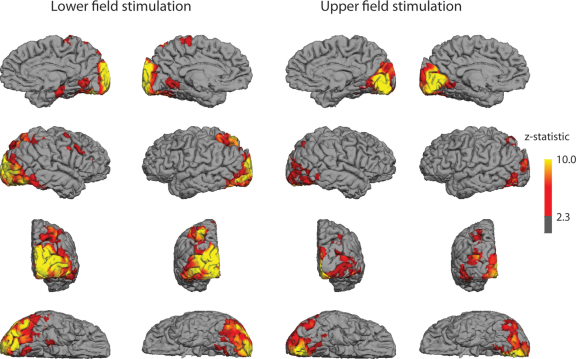


**Fig. 1:**
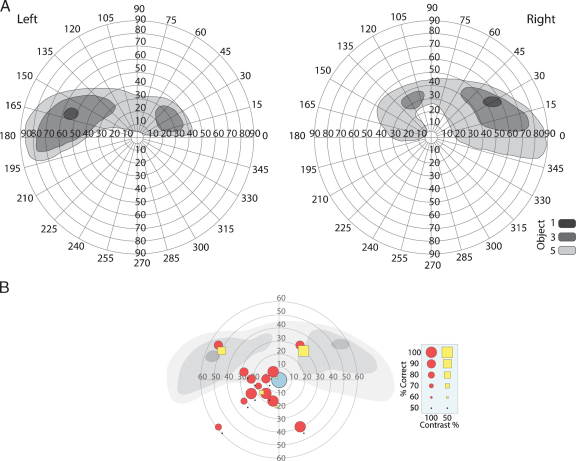
(A) Visual fields measured with Goldman perimetry. The largest region of residual visual function is in the upper peripheral field. Note that the fields are drawn with high contrast stimuli of different sizes all at brightest intensity. (B) The locations of the Gabor patches used for behavioral testing and the detection rates at each point, superimposed on the sum of the monocular visual fields from the perimetry. The blue circle indicates the mean fixation position (radius = 95% confidence interval), red circles indicate stimulus detection rates at 100% and yellow squares are detection rates at 50% contrast. (For interpretation of the references to color in this figure legend, the reader is referred to the web version of the article.)

**Fig. 2:**
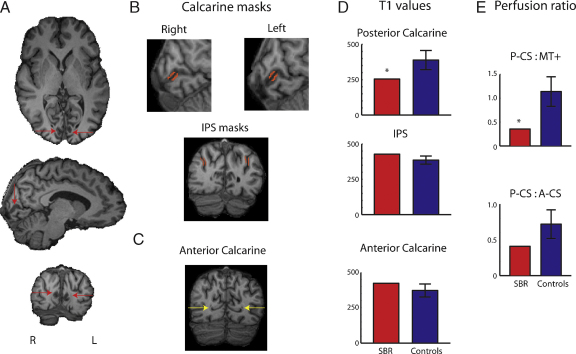
The gray matter of the posterior calcarine sulcus has a lower intensity than the surrounding gray matter (A). (B) The single voxel thickness masks used to compare the intensity of gray matter voxels in the calcarine and intra-parietal sulci. The very anterior region of spared CS is indicated with the yellow arrows (C). The extracted values from the T1-weighted image for each of these regions are shown in (D). The ratio of perfusion in the posterior CS (P-CS) compared to MT+/V5 and the anterior CS (A-CS) is shown in (E), indicating decreased perfusion to the posterior CS in SBR. Error bars show standard deviations and asterisk indicates SBR's data lying more than 2 standard deviations from the control mean.

**Fig. 3:**
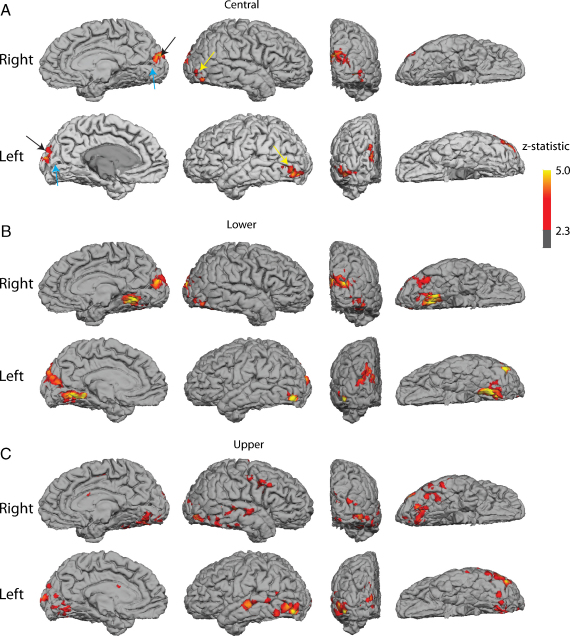
Activation in SBR to chequerboard stimuli placed (A) centrally, (B) in the lower and (C) in the upper visual field. In each case, there is bilateral activation of V5/MT (yellow arrows). There is V2/V3 activation when the stimulus is in the central and lower visual field, but not upper as would be predicted normally (black arrows). The calcarine sulcus itself (blue arrows) shows no significant activation on either bank in any condition. The ventral visual stream shows significant activation when the larger stimuli are used (Lower and Upper), but nothing for the smaller, central stimulus. The scale bar shows the significance of the activation, thresholded at *z* > 2.3, corrected.

**Fig. 4:**
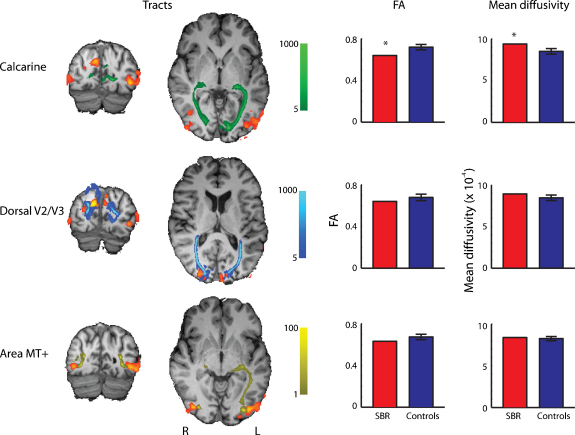
Left side shows tracts between the LGN and the Calcarine Sulcus, Dorsal V2/V3 and area MT+ in patient SBR. The orange/red regions are the areas activated by the central flashing chequerboard. The color bars in the centre of the figure indicate the probability of connectivity between the LGN and the three visual areas. The right side shows the FA and mean diffusivity for SBR and the controls in the white matter of each tract. Error bars show standard deviations and asterisk represents SBR's data lying more than 2 standard deviations from the control mean.
