# Erratum to “Visual activation of extra-striate cortex in the absence of V1 activation” [Neuropsychologia 48/14 (2010) 4148–4154]

**DOI:** 10.1016/j.neuropsychologia.2011.02.043

**Published:** 2011-04

**Authors:** Holly Bridge, Stephen L. Hicks, Jingyi Xie, Thomas W. Okell, Sabira Mannan, Iona Alexander, Alan Cowey, Christopher Kennard

**Affiliations:** a FMRIB Centre, University of Oxford, John Radcliffe Hospital, Oxford OX3 9DU, United Kingdom; b Department of Clinical Neurology, University of Oxford, John Radcliffe Hospital, Oxford OX3 9DU, United Kingdom; c Department of Experimental Psychology, University of Oxford, Parks Road, Oxford OX1 3UD, United Kingdom

The authors would like to apologise for an omission with regard to the funding received by one of the co-authors of the above paper. The following acknowledgement was omitted from the original publication in error:

‘C. Kennard was supported by the NIHR Biomedical Research Centre, Oxford.’

